# Factors Affecting Glycemic Control among Saudi Children with Type 1 Diabetes Mellitus in Aseer Region, Southwestern Saudi Arabia

**DOI:** 10.3390/ijerph191811558

**Published:** 2022-09-14

**Authors:** Saleh M. Al-Qahtani, Ayed A. Shati, Youssef A. Alqahtani, Ali A. AlAsmari, Mohammed A. Almahdi, Amjad A. Al Hassan, Ali M. Alhassany, Rana A. Shathan, Rawa M. Aldosari, Abdullah S. AlQahtani, Shamsun Nahar Khalil

**Affiliations:** 1 Department of Child Health, College of Medicine, King Khalid University, Abha 62223, Saudi Arabia; 2 Department of Pediatrics, Abha Maternity and Children Hospital, Abha 62562, Saudi Arabia; 3 Department of Pediatrics, Armed Forces Hospital-Southern Region, Khamis Mushyate 62413, Saudi Arabia; 4 Department Medicine, King Fahad Hospital, Al-Baha 65732, Saudi Arabia; 5 Department of Pediatric Emergency, Abha Maternity and Children Hospital, Abha 62562, Saudi Arabia; 6 Department of Emergency Medicine, Khamis Mushyate General Hospital, Khamis Mushyate 62433, Saudi Arabia; 7 Department of Family & Community Medicine, College of Medicine, King Khalid University, Abha 62223, Saudi Arabia

**Keywords:** type 1 diabetes mellitus, glycated hemoglobin, glycemic control, prevalence, associated factors

## Abstract

Glycemic control in children with type 1 diabetes mellitus (T1DM) is affected by many factors that may be influenced by their lives and community. To identify the factors associated with glycemic control among children with T1DM in Aseer Region, southwestern Saudi Arabia, a cross-sectional interview study was conducted between 1 July and 30 September 2021, with a representative sample of Saudi children aged between 6 months and 15 years with T1DM or their caregivers visiting the diabetes center at Aseer Region. Newly diagnosed cases (<12 months) were excluded from the study. The study included 171 T1DM pediatric patients aged between 18 months and 15 years. The glycated hemoglobin (HbA1c) level ranged between 6.10% and 15.10% (mean HbA1c = 10.39% ± 1.86%). High HbA1c levels (≥7.5%) were observed in most patients (94.7%). Only two significant factors were found: (1) use of carbohydrate counting; 81.8% of children using carbohydrate counts had high HbA1c levels, compared to 96.6% of children not using carbohydrate counts (*p* = 0.017), and (2) duration of the disease; 91.5% of children with disease duration of ≤3 years had high levels of HbA1c, compared to 98.7% of children with disease duration exceeding 3 years (*p* = 0.035). Most children with T1DM in Aseer Region had poor glycemic control. Only two factors were associated with better glycemic control: shorter disease duration and use of carbohydrate counting. Therefore, advising diabetic patients to be on a carbohydrate counting program might improve DM control.

## 1. Introduction

Type 1 diabetes mellitus (T1DM) in children is a major health problem worldwide and is considered the most common endocrine disorder [1]. The prevalence of T1DM has increased rapidly over the last three decades, with 190 per 100,000 among school-aged children in the USA [2] and 109.5 per 100,000 in Saudi Arabia [3]. The annual incidence of T1DM is 31.4 per 100,000 according to the International Diabetes Federation’s Diabetes Atlas [4].

The most important marker of control of T1DM is the stability and consistency of maintaining normal blood glucose levels, usually described as glycemic control. The use of hemoglobin A1c (HbA1c) is a well-established marker of glycemic control. A HbA1c value of less than 7.5% is regarded as good control of blood sugar levels, while patients with HbA1c levels of 7.5% or more are considered to have poor glycemic control [5,6]. The Diabetic Control and Complication Trial (DCCT) confirmed the role of blood glucose control in reducing the risk of T1DM complications [7]. The ability to achieve satisfactory control is the main factor in avoiding future complications, such as organ damage. Poor glycemic control, including both hyper and hypoglycemia, has been associated with a higher risk of in hospital deaths [8]. In Jeddah, Saudi Arabia, Al-Agha et al. (2015) confirmed that poor glycemic control was the leading cause of diabetic ketoacidosis [9].

Current literature states that depression is increasingly more common in individuals suffering from diabetes. Many of the core symptoms of depression, including poor diet and sleep, lack of physical activity, and reduced compliance with medication, may very well contribute to poor control of diabetes [10]. Links between diabetes and depression have been mentioned previously [11]. High levels of circulating cytokines associated with both conditions leads to insulin deficiency, impaired neurogenesis and neurotransmitter metabolism. Cardiovascular risks are also higher in patients with diabetes as poor glycemic control is associated with abdominal obesity and hyperlipidemia [12,13].

Most chronic conditions including T1DM can have a great impact on the lives of children living with the condition. Children with T1DM may have many factors that contribute to issues with glycemic control, such as age, ethnicity, poor compliance with insulin therapy, and obesity. Other factors related to the diagnosis and treatment, such as duration of living with diabetes, the type of insulin therapy and access to advice and support, family support, and the psychological effects of living with a chronic condition whilst growing up, can also pose many issues to children trying to achieve good glycemic control [14,15].

There is a lack of studies that identify and correlate causes of poor glycemic control in children. Clear understanding of the factors determining satisfactory glycemic control in children is vital to allow patients to control their condition and avoid any related complications.

Thus, the current study aims to identify factors affecting glycemic control among children with T1DM in Aseer Region, Saudi Arabia. This will provide an insight into the current assessment of glycemic control in children in this region, and also provide an opportunity to highlight any difficulties or hurdles that may exist and impair children trying to attain better control of their condition and hence avoid any related complications.

## 2. Methods

A cross-sectional interview study was conducted between 1 July and 30 September 2021, with a representative sample of 171 children with T1DM and their caregivers visiting the diabetes center in Aseer Region, Saudi Arabia. 

The inclusion criteria were children of Saudi nationality aged younger than 15 years. Newly diagnosed cases (<12 months) were excluded from the study. A consecutive sampling technique was adopted to include Saudi children with T1DM visiting the diabetes center until the sample was completed.

Children with T1DM and their caregivers were enrolled by a nurse. The attending nurse introduced the data collectors to the patients and their family members. Data were collected by face-to-face interviews with the family members in the waiting area. Written consent was obtained from those who participated in the interview prior to their inclusion, and the consent was written in Arabic.

A questionnaire was developed with intensive literature review on the topic. The questionnaire comprised of demographic data including age at diagnosis, caregiver, current parental marital status, family income, housing type, and parental education. In addition, the questionnaire included questions related to the disease and its management, including the duration of disease, insulin type, dosage, history of hospitalization, intensive care unit (ICU) admission, use of carbohydrate counting, diet, physician revisit interval, adherence to insulin therapy, family knowledge about glucagon, glucagon injection, availability of diabetes educators, the sufficiency of information from diabetes educators, and availability of glucose test devices. Blood glycosylated hemoglobin (HbA1c) is used as an indicator of glycemic control. In this study, the patients were said to have good glycemic control if they had HbA1c less than 7.5% and were said to have poor control if HbA1c was higher or equal to 7.5% [16]. Blood HbA1c measures were documented from the patient’s medical record.

This research study was approved by the Research Ethics Committee of the College of Medicine, King Khalid University (REC#14-4-2017).

### Statistical Analysis

Data were analyzed using Statistical Package for Social Sciences, version 25.0 (SPSS, Inc., Chicago, IL, USA). Data were expressed using frequencies and percentages; analyses were performed using the chi-square test to assess the association between diabetes control and its possible associated factors. Fischer’s exact test was applied instead of the chi-square test in cases where the expected frequency was <5 in one or more of the cells. The level of statistical significance was *p* < 0.05.

## 3. Results

**Figure 1:**
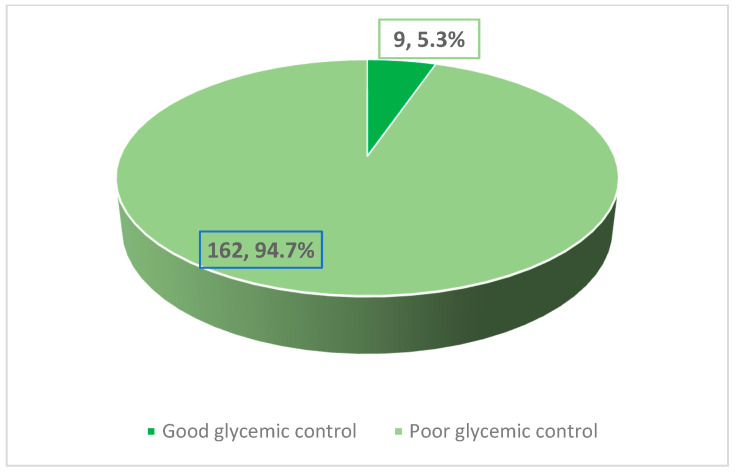
Prevalence of uncontrolled type 1 diabetes mellitus among children in the Aseer Region.

The study included 171 pediatric patients with T1DM. Their age ranged between 18 months and 15 years. Approximately 53.8% of the patients were males (*n* = 92). The glycated hemoglobin (HbA1c) levels ranged between 6.10% and 15.10%, with a mean and standard deviation (SD) of 10.39% ± 1.86%. As observed in Figure 1, the majority of the patients (94.7%) had high levels of HbA1c (≥ 7.5%).

As shown in Table 1, none of the studied socio-demographic factors (age at diagnosis, gender, current parental marital status, caregiver, educational level of caregivers, family income, and housing type) were significantly associated with T1DM control, manifested by the HbA1c level.

Among the studied factors related to diabetes, only diabetes management and disease duration (Table 2) appeared to be significant: (1) use of carbohydrate counting; approximately 81.8% of children who used carbohydrate counts had high HbA1c levels (≥7.5%), compared to 96.6% of children who did not (*p* = 0.017); and (2) duration of disease; roughly 91.5% of children with a disease duration of 3 years or less had high HbA1c levels (≥7.5%), compared to 98.7% of those with a disease duration exceeding 3 years (*p* = 0.035).

## 4. Discussion

Identifying children with T1DM at high risk for poor glycemic control is essential to implementing aggressive interventions and disease management, and to prevent further deterioration of glycemic control. For this purpose, physicians need to identify these children to ensure optimum preventative care [17]. Therefore, this study was conducted to identify factors associated with glycemic control among children with T1DM in Aseer Region, KSA.

Overall, our findings were consistent with previous studies [18,19,20,21,22] reporting poor glycemic control, as evidenced by HbA1c levels. Among the variables studied in the present study, only two were significant: duration of diabetes mellitus and the use of carbohydrate counting by caregivers. In accordance with previous studies [18], our results showed that the duration of T1DM was significantly associated with HbA1c level. Similar to the findings reported by Yazidi et al., [18], we observed that HbA1c levels were the lowest during the first year of the disease but increased significantly thereafter. Indeed, the honeymoon phenomenon occurs in the first month of disease diagnosis and is characterized by the presence of few beta cells capable of producing intrinsic insulin [23].

Our finding that the use of carbohydrate counting was significantly associated with better glycemic control is corroborated with findings reported by Mehta et al. [24]. Moreover, other older studies have reported that the use of carbohydrate counting was independently associated with lower HbA1c levels [25,26,27]. Carbohydrate counting leads to proper insulin calculation, thereby improving glycemic control [24]. In a randomized controlled trial, Laurenzi et al. [27] concluded that patients with T1DM using continuous subcutaneous insulin infusion with carbohydrate counting showed reduced HbA1c levels. Tascini et al. [28] concluded that the use of carbohydrate counting might reduce HbA1c concentrations. Similarly, a recent systematic review and meta-analysis reported similar findings [29].

Although the present study does not show any effect of the age at diagnosis on HbA1c level, Yazidi et al. [18] reported that a young age at diagnosis was associated with higher HbA1c levels during follow-up due to faster autoimmune β-cell destruction. Similarly, in a large cohort of patients with T1DM, Clements et al. observed that old age at diagnosis was significantly associated with poor glycemic control [17].

In accordance with another study [9], the child’s gender was not a predictor for poor glycemic control in the present study. However, several studies have reported that girls with T1DM had worse metabolic control than boys and attributed this to a higher insulin resistance [23,30,31].

Regarding insulin treatment, in accordance with Yazidi et al. [18], our study showed that insulin dosage was not significantly associated with glycemic control. However, the Diabetes Control and Complication Trial (DCCT) reported that intensive insulin therapy in the form of continuous subcutaneous insulin infusion, or three or more injections per day, resulted in better glycemic control than conservative therapy in the form of two injections per day [32]. Several studies have reported a significant association between non-adherence to insulin therapy and poor glycemic control [18,19,33,34]. However, we could not confirm this finding in the current study due to our relatively small sample size.

In this study, we found that the physician revisit interval was not significantly related to glycemic control. Our finding was contrary to those reported previously [18,35,36], all of which showed that the number of clinic visits per year contributed to better glycemic control. In a study conducted in Brazil [19], self-reported poor adherence to the diet was strongly associated with higher levels of HbA1c. In the current study, we failed to find an association between diet and levels of HbA1c.

In previous studies conducted in Brazil [19], Australia [37], and Saudi Arabia [38], participation in a diabetes education program was significantly associated with better glycemic control. However, in the present study, the availability of diabetes educators and the opportunity to learn from diabetes educators were not significantly associated with glycemic control. One potential explanation may be related to the quality of training and information provided by these diabetes educators.

Some limitations of this study should be taken into consideration. First, the study was conducted in one region of Saudi Arabia, which could affect the generalizability of the results. Second, the relatively small number of cases with good control could affect the possibility of significant associations. Third, the cross-sectional nature of our study does not allow for the establishment of a temporal relationship between HbA1c levels and possible associated factors. Finally, the data, with the exception of HbA1c level data, were collected through interviewing caregivers, and thus may be prone to information bias.

## 5. Conclusions

The majority of children with T1DM in Aseer Region, Saudi Arabia, had poor glycemic control. Only two factors were associated with better glycemic control: shorter duration of disease and the use of carbohydrate counting. For this reason, more efforts are needed to increase awareness among physicians dealing with children with T1DM and their families regarding the role of carbohydrate counting in improving glycemic control. Further research with a larger sample size focusing on the use of carbohydrate counting in children with T1DM is needed.

## Figures and Tables

**Table 1:** Socio-demographic factors associated with level of HbA1c among children in Aseer Region.

	HbA1C		*p*-Value
	Good Glycemic Control *n* = 9 *n* (%)	Poor Glycemic Control *n* = 162 *n* (%)	
**Gender** Male (n 92) Female (n 79)	7 (7.6) 2 (2.5)	85 (92.4) 77 (97.5)	0.127 *
**Current parental marital status** Married (*n* = 166) Unmarried (*n* = 5)	8 (4.8) 1 (20.0)	156 (95.2) 4 (80.0)	0.239 *
**Caregiver** Mothers only (*n* = 128) Both parents (*n* = 24) Patient self-care (*n* = 14) Others (*n* = 5)	7 (5.5) 0 (0.0) 2 (14.3) 0 (0.0)	121 (94.5) 24 (100) 12 (85.7) 5 (100)	0.272 **
**Educational level of the caregiver** Illiterate (*n* = 6) Elementary school (*n* = 42) Intermediate school (*n* = 20) High school (*n* = 38) University (*n* = 65)	0 (0.0) 4 (9.5) 0 (0.0) 2 (5.3) 3 (4.6)	6 (100) 38 (90.5) 20 (100) 36 (94.7) 62 (95.4)	0.553 **
**Family income (SR/month)** <5000 (*n* = 15) 5000–10,000 (*n* = 76) 10,001 < 15,000 (*n* = 57) ≥15,000 (*n* = 23)	1 (6.7) 5 (6.6) 3 (5.3 0 (0.0)	14 (93.3) 71 (93.4) 54 (94.7) 23 (100)	0.6599 **
**Housing type** Private (*n* = 120) Rented (*n* = 51)	6 (5.0) 3 (5.9)	114 (95.0) 48 (94.1)	0.536 **
**Age at diagnosis (years)** <3 (*n* = 32) 3–6 (*n* = 65) >6 (*n* = 74)	2 (6.3) 2 (3.1) 5 (6.8)	30 (93.8) 63 (96.9) 69 (93.2)	0.601 **

* Fischer exact test; ** Chi-square test.

**Table 2:** Diabetes mellitus related factors associated with level of HbA1c among children in Aseer Region.

	HbA1C		*p*-Value
	Good Glycemic Control *n* = 9 *n* (%)	Poor Glycemic Control *n* = 162 *n* (%)	
**Duration of DM (years)** ≤3 (*n* = 94) >3 (*n* = 77)	8 (8.5) 1 (1.3)	86 (91.5) 76 (98.7)	0.035 *
**History of hospitalization** No (*n* = 59) Once (*n* = 61) Twice (*n* = 35) >Twice (*n* = 16) ICU admission Yes (*n* = 53) No (*n* = 118)	1 (1.7) 5 (8.2) 2 (5.7) 1 (6.3) 4 (7.5) 5 (4.2)	58 (98.3) 56 (91.8) 33 (94.3) 15 (93.7) 49 (92.5) 113 (95.8)	0.457 ** 0.290 *
**Carbohydrate count usage** (*n* = 170) Yes (*n* = 22) No (*n* = 148)	4 (18.2) 5 (3.4)	18 (81.8) 143 (96.6)	0.017 *
**Diet usage** Yes (*n* = 35) No (*n* = 136)	2 (5.7) 7 (5.1)	33 (94.3) 129 (94.9)	0.583 *
**Physician revisit interval** Once/month (*n* = 8) Once/3 months (*n* = 150) Once/6 months (13)	0 (0.0) 9 (6.0) 0 (0.0)	8 (100) 141 (94.0) 13 (100)	0.514 **
**Adherence to insulin therapy** Excellent (*n* = 117) Good (*n* = 52) Bad (*n* = 2)	7 (6.0) 2 (3.8) 0 (0.0)	110 (94.0) 50 (96.2) 2 (100)	0.802 **
**Type of insulin** Multi-dosage/day (*n* = 105) Two doses/day (*n* = 62) Insulin pump (*n* = 4)	6 (5.7) 3 (4.8) 0 (0.0)	99 (94.3) 59 (95.2) 4 (100)	0.866 **
**Knowledge of the family about glucagon** Yes (*n* = 153) No (*n* = 18)	7 (4.6) 2 (11.1)	146 (95.4) 16 (88.9)	0.242 *
**Glucagon injection** Yes (*n* = 151) No (*n* = 20)	8 (5.3) 1 (5.0)	143 (94.7) 19 (95.0)	0.716 **
**Availability of diabetes educator** Yes (*n* = 141) No (*n* = 13) Do not know (*n* = 17)	7 (5.0) 1 (7.7) 1 (5.9)	134 (95.0) 12 (92.3) 16 (94.1)	0.908 **
**Sufficiency of information from a diabetes educator** Yes (*n* = 133) No (*n* = 37)	7 (5.3) 2 (5.4)	126 (94.7) 35 (94.6)	0.622 *
**Availability of glucose test device** No (*n* = 2) Yes, from the government (*n* = 140) Yes, on patient’s exposure (*n* = 29)	0 (0.0) 8 (5.7) 1 (3.4)	2 (100) 132 (94.3) 28 (96.6)	0.835 **

* Fischer exact test; ** Chi-square test.

## Data Availability

The datasets used and analyzed in the current study are available from the corresponding author on reasonable request.
